# Current status of sleep quality in Taiwan: a nationwide walk-in survey

**DOI:** 10.1186/s12991-015-0078-7

**Published:** 2015-11-02

**Authors:** Shu-Yu Tai, Wen-Fu Wang, Yuan-Han Yang

**Affiliations:** Department of Family Medicine, Kaohsiung Municipal Ta-Tung Hospital, Kaohsiung, Taiwan; Department of Family Medicine, Kaohsiung Medical University Hospital, Kaohsiung, Taiwan; Graduate Institute of Medicine, College of Medicine, Kaohsiung Medical University, Kaohsiung, Taiwan; Department of Family Medicine, School of Medicine, College of Medicine, Kaohsiung Medical University, Kaohsiung, Taiwan; Department of Neurology, Changhua Christian Hospital, Changhua, Taiwan; Department of Neurology, Kaohsiung Municipal Ta-Tung Hospital, Kaohsiung, Taiwan; Department of Neurology, Kaohsiung Medical University Hospital, Kaohsiung, Taiwan; Department of and Master’s Program in Neurology, Faculty of Medicine, Kaohsiung Medical University, Kaohsiung, Taiwan

**Keywords:** Walk-in screen, Sleep quality, PSQI, Hypnotics, Taiwan

## Abstract

**Background:**

Sleep disorder plays an important role in the overall health care system, because it can be co-morbid with many other physical or mental disorders. In this study, we conducted a screening survey to determine the current status of sleep quality in the general population of Taiwan.

**Methods:**

During the period 1 March 2010 to 30 April 2013, we collaborated with the Fo-Guang Shan Compassion Foundation’s Mentality Protection Center (MPC) branches to conduct 53 walk-in screenings at the 59 branches distributed throughout Taiwan. We used the Pittsburgh Sleep Quality Index (PSQI) to assess multiple dimensions of sleep over a 1-month period after identifying the participants’ age, sex, and residence location. The participants were identified to have poor sleep quality if their PSQI-T total score was greater than five.

**Results:**

In total, 760 participants, 195 from northern, 289 from central, 228 from southern, and 48 from eastern Taiwan with an average age of 54.2 years (SD 14.7) were recruited. We found that 46.6 % of all participants had poor sleep quality and that there were significantly different proportions among the four areas. Besides, 11.6 % of all participants and 21.8 % of individuals with poor sleep quality had used sedatives/hypnotics to help them fall asleep in the past 4 weeks, and the proportion was highest in the eastern area.

**Conclusions:**

This survey suggested that the ratio of poor sleep quality in Taiwan is progressively increasing compared to the previous studies. In addition, there were significantly different proportions of individuals with poor sleep quality and hypnotics’ uses among the four areas.

## Background

Sleep disorders are problems of the sleeping patterns, and broadly classified into four main types, including problems of falling and staying asleep, staying awake, sticking to a regular sleep schedule, and unusual behaviors during sleep [1]. An estimated 25–30 % of the general adult population and a comparable percentage of children and adolescents chronically suffer from sleep disorders that are proven contributors to disability, morbidity, and mortality [2]. Sleep disorder can occur as a primary disorder or, more commonly, it can be co-morbid with other physical or mental disorders [3]. When a person suffers from difficulty falling asleep and/or staying asleep with no obvious cause, it is referred to as insomnia [4]. Around 50 % of patients with depression have co-morbid insomnia, and depression and sleep disturbance are, respectively, the first and third most common psychological reasons for patient encounters in general practice [5]. Insomnia doubles the risk of future development of depression [6] and increases the risk of a range of health concerns and chronic diseases, including substance abuse, poor daytime functioning [7], dissatisfaction with one’s quality of life [8], obesity [9], diabetes mellitus [10], and hypertension [6, 11]. These associations contributed to a significant economic burden, and annual sleep disorder-related direct and indirect cost estimates exceed $100 billion in the United States [12] and $40 million in Taiwan [13]. Understanding the situation of sleep is an essential first step in establishing public health priorities for preventing sleep-related health concerns.

Although information regarding the national sleep disorder prevalence rate is lacking in Taiwan, two cross-sectional surveys of insomnia, the most common sleep disorder, in Asian countries were conducted in 2003 [14] and 2005 [15]. The cross-sectional survey with a face-to-face interview in 2003 defined insomnia as having at least one of three types of insomnia symptoms (i.e., difficulty initiating sleep, difficulty maintaining sleep, and early-morning awakening), The study revealed that the age-adjusted prevalence of insomnia was 10.3 % in Taiwan, which was higher than that in Japan (4 %) and Korea (9.9 %) [14]. Another nationwide data of 36,743 men and women aged 18 years and above from the 2005 Survey of Social Development Trends-Health Security in Taiwan were analyzed. This 2005 survey used the insomnia self-assessment inventory which is a 13-item self-reported inventory designed by the WHO worldwide project on sleep and health to measure insomnia and found that over 25 % of Taiwanese adults’ experienced insomnia [15]. Although these two studies were both nationwide survey, they were published several years ago [14, 15]. Other prior studies were limited to a specific age group (i.e., children [16], adolescents [17] or elderly [18]), gender [19], or medical condition [20].

A recent eight-year nationally representative study used the National Health Insurance database to assess the longitudinal healthcare-seeking prevalence and incidence of insomnia with ICD-9 of 780.52, 307.41, and 307.42 [21]. The result revealed that the prevalence of insomnia was 5.4 % for women and 3.0 % for men in 2009; the incidence of insomnia was 1.6 % for women and 1.3 % for men. Although this was a recent nationwide population-based cohort study, it reflected the prevalence and incidence of health-seeking behavior and diagnosis of insomnia and was not the prevalence of symptoms or a sleep complaint. Screening, in medicine, is an important strategy used in a population to identify an unrecognized disease in individuals without signs or symptoms. Therefore, we conducted this screening survey to determine the current status of sleep quality in the general population in Taiwan.

## Methods

### Study population

The Mentality Protection Center (MPC) is a non-profit institution that was established under the Fo-Guang Shan Compassion Foundation in 2008 to provide medical and charitable services to the general population worldwide through the hundreds of Fo-Guang Shan branches. There are 59 MPC branches in Taiwan. The branches are scattered throughout the northern, central, southern, and eastern parts of Taiwan and are distributed in urban, suburban, and rural areas in each part of Taiwan.

### Study sample

During the period 1 March 2010 to 30 April 2013, we collaborated with the Fo-Guang Shan branches in conducting 53 walk-in screenings among the 59 branches of MPC. Each screening at a branch lasted 1 day and provided medical and charitable services to the general population, including screening for dementia using the ascertainment of dementia 8(AD8) [22], depression using the Center for Epidemiologic Studies-Depression (CES-D) scale [23], and sleep quality using the Pittsburgh Sleep Quality Index (PSQI) [24]. Seven of the 53 screenings of the general population were conducted in northern Taiwan, 19 in the central area, 24 in the south, and 3 in the east.

### Screening instrument for sleep quality

The Pittsburgh Sleep Quality Index (PSQI) is a self-report questionnaire that assesses multiple dimensions of sleep over 1-month period [25, 26]. Nineteen individual items were used to generate seven ‘component’ scores: subjective sleep quality, sleep latency, sleep duration, habitual sleep efficiency, sleep disturbances, use of sleeping medication, and daytime dysfunction. The sum of the seven component scores yields one global score of subjective sleep quality (range 0–21); higher scores represent poorer subjective sleep quality. The Taiwanese version of the Pittsburgh Sleep Quality Index (PSQI-T) [27] has good validity and reliability using the cut-off point of five to determine the “poor sleep quality” status.

### Participants and evaluation

All participants were volunteers who took part in the screening activity without any reward. The CES-D, AD8, and PSQI were administered to participants after identifying their age, sex, and residence location. The participant had poor sleep quality if his/her PSQI-T total score was greater than five. All procedures were approved by the Kaohsiung Medical University Hospital Institutional Review Board (KMUH-IRB-20140054). Since all identifying personal information was not recorded during the screening process prior to analysis, the review board waived the requirement for written informed consent from the patients involved.

### Use of hypnotics

The frequency of use of hypnotics was quantified on the basis of an additional question not derived from the PSQI, and that specifically inquired “During the last 4 weeks, on how many nights did you use sedatives/hypnotics to help fall sleep?” Participants who reported ≥one instances of use of sedatives/hypnotics within the last 4 weeks were identified as “sedatives/hypnotics users.”

### Sleep duration

Sleep duration was estimated from responses to the following self-report question: “During the past month, how many hours of actual sleep did you get at night?” derived from the PSQI. Sleep duration was categorized in 1-h units.

### Statistics

Data analysis was performed using SPSS (version 12.0.1 for Windows, SPSS Inc., Chicago, IL, USA). All statistical tests were two-tailed, and the alpha that indicates significance was <0.05. Analysis of variance (ANOVA) was used to compare the differences in group mean for age, sleep duration, PSQI total score, and subscale score among the four areas (northern, central, southern, and eastern) of Taiwan for all recruited participants and for poor-sleep-quality individuals. The *T* test was used to compare the group mean of age, sleep duration, PSQI total score, and subscale score of PSQI between poor and good sleep quality individuals. The Chi square test was used to compare the proportion of gender and hypnotics use between the poor and good sleep quality individuals and among the four areas of Taiwan.

## Results

In total, 760 participants, 195 in northern, 289 in central, 228 in southern, and 48 in eastern Taiwan, with an average age of 54.2 years (SD 14.7), were recruited. The average age was significantly different among the four areas ( < 0.001). The participants in eastern Taiwan were older (mean (SD) age: 70.1 (9.6) years) than those in the other three areas (Table 1). Participants were predominantly females and the gender proportion was significantly different among the four areas ( < 0.001); it was higher in the northern area (93.6 %), compared to the other areas (Table 1). Among the total population, the average nighttime sleep duration and PSQI score were 6.2 h (SD 1.4) and 6.1 points (SD 3.6), respectively, with significant differences between the four areas ( < 0.001). The longest average sleep duration was 6.5 h (SD 1.4) in the eastern area and the shortest duration was 5.6 h (SD 1.3) in the northern area; the highest average PSQI score was 7.0 (SD 3.5) in the northern area and the lowest score was 5.6 (SD 4.4) in the eastern area. Approximately 12 % of all participants had used sedatives/hypnotics to help them fall asleep in the past 4 weeks and the proportion was highest in the eastern area (25.0 %) (Table 1).

**Table 1:** Demographic characteristics of all participants (N = 760)

	Northern	Middle	Southern	Eastern	*P* value	Total
N (%)	195 (25.7)	289 (38.0)	228 (30.0)	48 (6.3)		760 (100)
Age (years)^a^						
Mean ± SD	45.5 ± 12.1	53.5 ± 13.5	59.8 ± 14.6	70.1 ± 9.6	*<0.001* ^§^	54.2 ± 14.7
Gender (n, %)					*<0.001* ^§^	
Men	12 (6.5)	82 (29.8)	43 (19.0)	20 (41.7)		157 (21.4)
Women	174 (93.6)	193 (70.2)	183 (81.0)	28 (58.3)		578 (78.6)
Sleep duration (hours)^b^						
Mean ± SD	5.6 ± 1.3	6.4 ± 1.2	6.3 ± 1.4	6.5 ± 1.4	*<0.001* ^§^	6.2 ± 1.4
Hypnotics use (days/4 weeks)					*<0.001 * ^§^	
No use	184 (94.4)	257 (88.9)	195 (85.5)	36 (75.0)		672 (88.4)
1–2	4 (2.0)	13 (4.5)	14 (6.1)	1 (2.1)		32 (4.2)
≥3	7 (3.6)	19 (6.6)	19 (8.4)	11 (22.9)		56 (7.4)
PSQI^#^score^c^					*<0.001* ^§^	
Mean ± SD	7.0 ± 3.5	5.8 ± 3.4	5.7 ± 3.7	5.6 ± 4.4		6.1 ± 3.6

^#^PSQI score: Pittsburgh Sleep Quality Index score

^§^ *P* value < 0.05

^a^Four areas are significantly different

^b, c^The northern area is significantly different from other areas

A total of 46.6 % of all participants had poor sleep quality with PSQI scores greater than five. There were similar average age (*P* = 0.221) and distribution of gender (*P* = 0.064) among the two groups, although there was significant different of the PSQI score between genders (The mean (SD) of PSQI was 6.02(3.42) in 578 females and 5.13(3.37) in 157 males; the *P* value was 0.004). The tests for between-group comparisons of the PSQI scores and seven subscale scores were all significant (all *P*s < 0.001). Participants in the poor-sleep-quality group had 5.5-h (SD 1.4) average sleep duration, which was shorter than that in the good sleep quality group [6.8 h (SD 1.0)]. A significantly different proportion of participants in the poor (21.8 %) and good (2.7 %) sleep quality groups used sedatives/hypnotics to help fall asleep (Table 2).

**Table 2:** Comparison and distribution of the Pittsburgh Sleep Quality Index (PSQI) scores among poor and good sleep quality groups

	Good sleep quality^a^	Poor sleep quality^a^	*P* value
N, %	406, 53.4 %	354, 46.6 %	
Age (years)			0.221
Mean ± SD	54.8 ± 14.1	53.5 ± 15.3	
Gender (n, %)			0.064
Men	94 (59.9)	63 (40.1)	
Women	298 (51.6)	280 (48.4)	
Sleep duration (hours)			*<0.001* ^§^
Mean ± SD	6.8 ± 1.0	5.5 ± 1.4	
Hypnotics use (days/4 weeks)			*<0.001* ^§^
No use	395 (97.3)	277 (78.2)	
1–2	9 (2.2)	23 (6.5)	
≥3	2 (0.5)	54 (15.3)	
PSQI score^b^			*<0.001* ^§^
Mean ± SD	3.4 ± 1.3	9.1 ± 2.9	
Subscale score: mean (SD)			
Subjective sleep quality	0.5 (0.6)	1.5 (0.8)	*<0.001* ^§^
Sleep latency	0.6 (0.6)	1.7 (0.9)	*<0.001* ^§^
Sleep duration	1.0 (0.8)	1.8 (0.9)	*<0.001* ^§^
Habitual sleep efficiency	0.2 (0.4)	1.1 (1.2)	*<0.001* ^§^
Sleep disturbances	0.9 (0.4)	1.3 (0.6)	*<0.001* ^§^
Use of sleeping medication	0.0 (0.3)	0.6 (1.2)	*<0.001* ^§^
Daytime dysfunction	0.2 (0.5)	1.1 (0.8)	*<0.001* ^§^

^§^ *P *value < 0.05

^a^Good sleep quality: PSQI ≤ five; poor sleep quality: PSQI > five

^b^PSQI score: Pittsburgh Sleep Quality Index score

The proportion of poor-sleep-quality participants was significantly different among the four areas. The highest proportion was in the northern area (57.9 %) and lowest was in the eastern area (37.5 %). The distribution of gender among poor-sleep-quality individuals in the four areas was significantly different. The highest proportion of females was in the northern area (94.6 %) and the lowest was in the eastern area (61.1 %). The average age (*P* < 0.001) and sleep duration (*P* = 0.002) among the four areas were significantly different, but the PSQI score (*P* = 0.149) was not. Among poor-sleep-quality participants, 21.8 % had used sedatives/hypnotics to help them fall asleep in the past 4 weeks and the proportions were significantly different among the four areas. The proportion was highest in the eastern area (66.7 %) and lowest in the northern area (8.8 %). Participants with poor sleep quality among the four areas had significantly different scores in all subscales except ‘subjective sleep quality’ and ‘habitual sleep efficiency’ (Table 3).

**Table 3:** Comparison and distribution of the Pittsburgh Sleep Quality Index (PSQI) scores of poor-sleep-quality participants in different areas (N = 354)

	Northern (N = 195)	Middle (N = 289)	Southern (N = 228)	Eastern (N = 48)	*P* value	Total (N = 760)
Number	113	132	91	18	*<0.001* ^§^	354
n, %	57.9 %	45.7 %	39.9 %	37.5 %		46.6 %
Age (years)^a^					*<0.001* ^§^	
Mean ± SD	44.4 ± 10.8	53.5 ± 14.3	62.1 ± 15.2	67.8 ± 9.1		53.5 ± 15.3
Gender (n, %)					*<0.001* ^§^	
Men	6 (5.4)	37 (29.6)	13 (14.4)	7 (38.9)		63 (40.1)
Women	104 (94.6)	88 (70.4)	77 (85.6)	11 (61.1)		280 (48.4)
Sleep duration (hours)^b^					*0.002* ^§^	
Mean ± SD	5.1 ± 1.3	5.7 ± 1.2	5.6 ± 1.7	5.8 ± 1.7		5.5 ± 1.4
Hypnotics use (days/4 weeks)					*<0.001* ^§^	
No use	103 (91.2)	105 (79.5)	63 (69.2)	6 (33.3)		277 (78.2)
1–2	3 (2.6)	8 (6.1)	11 (12.1)	1 (5.6)		23 (6.5)
≥3	7 (6.2)	19 (14.4)	17 (18.7)	11 (61.1)		54 (15.3)
PSQI score^#^					0.149	
Mean ± SD	9.2 ± 2.8	8.8 ± 2.7	9.2 ± 3.2	10.4 ± 3.6		9.1 ± 2.9
Subscale score: mean ± SD						
Subjective sleep quality	1.6 ± 0.6	1.5 ± 0.8	1.4 ± 0.9	1.8 ± 0.9	0.074	1.5 ± 0.8
Sleep latency	1.5 ± 0.8	1.7 ± 0.8	2.0 ± 0.9	2.1 ± 1.0	*<0.001* ^§^	1.7 ± 0.9
Sleep duration	2.1 ± 0.8	1.7 ± 0.9	1.7 ± 0.9	1.8 ± 1.0	*<0.001* ^§^	1.8 ± 0.9
Habitual sleep efficiency	1.1 ± 1.1	1.0 ± 1.2	1.3 ± 1.3	0.8 ± 1.1	0.378	1.1 ± 1.2
Sleep disturbances	1.4 ± 0.6	1.4 ± 0.6	1.3 ± 0.6	1.0 ± 0.3	*0.041* ^§^	1.3 ± 0.6
Use of sleeping medication	0.3 ± 1.0	0.5 ± 1.1	0.7 ± 1.2	1.9 ± 1.5	*<0.001* ^§^	0.6 ± 1.2
Daytime dysfunction	1.3 ± 0.9	1.0 ± 0.7	0.9 ± 0.8	1.0 ± 0.8	*0.004* ^§^	1.1 ± 0.8

^#^PSQI score: Pittsburgh Sleep Quality Index score

^§^ *P* value < 0.05

^a^The northern and middle areas are significantly different from other areas

^b^The northern area is significantly different from other areas

## Discussion

This study provides updated information on the current status of sleep quality in the general Taiwanese population and compares the difference among the four areas in Taiwan. In this study, we found that females were more incline to participate this walk-in screening, that may be also resulted from there is female predominant for people aged greater than 50 years old in Taiwan [28].

We also observed that the mean age of all recruited participants was significantly older in the eastern area than in the other areas of Taiwan. This may be related to the finding in the recent “Report on the Survey of Citizens’ Life Status in the Taiwan Area” published by the government, that the eastern area has a higher proportion of older persons than other areas. The government study reported that 11.5 % of the population in Taiwan is aged (>65 years old), with 10.8 % in the north, 11.8 % in the central area, 12.4 % in the south, and 13.4 % in the east, as of 31 December, 2013 [29]. Besides, the sleep duration was longer and the proportion of hypnotics used was greater in eastern Taiwan. Despite our new finding of a higher proportion of hypnotics use in eastern Taiwan, no updated related studies have addressed this issue. Further studies using randomized sampling are necessary to clarify these issues.

With regard to the prevalence of hypnotics use, 11.6 % of all participants, ranging from 5.6 % in the northern area to 25.0 % in the eastern area, used hypnotics. As for other countries, the prevalence of benzodiazepine use was 31.4 % in Chile [30], that of hypnotics use in Spain was 12.3 % [31], and in the USA, 6.2 % [32]. The wide variation in prevalence rates of hypnotics use reported in different countries can largely be explained by differences in definitions of hypnotics use, the healthcare system, the availability of hypnotics, and even the observation period.

This study found that 46.6 % (n = 354, total = 760) of the participants had a PSQI score greater than five, which was indicative of poor sleep quality. The prevalence of poor sleep quality among Taiwanese showed a progressive increase in this study compared to previous studies, which were conducted in the different period with the different definition of sleep disorder [14, 15]. In contrast to other studies [21, 33], which suggested that several demographic factors (e.g., female sex and increasing age) were associated with sleep problems, there were no similar results in this study. We also found that 21.8 % of the participants with poor sleep quality had used hypnotics to help fall asleep in the past 4 weeks. Hsu et al. conducted an eight-year nationally representative study and found that the prevalence of clinic-visiting behavior for sleep disorder was 5.4 % for women and 3.0 % for men in 2009 [21]. This inconsistency may be due to the fact that our data were based on the actual population prevalence rather than healthcare-seeking behavior, although healthcare-seeking behavior may be an indicator as this could represent a significant problem. However, Hsu et al. used the National Health Insurance database to assess the healthcare-seeking prevalence, which only included the western medicine. Moreover, the participants were not asked about the types of sedatives/hypnotics used in our study, and all agents were included in one category. Further detailed information on the definition of hypnotics is needed for clarity because Chinese herbal medicine or health (functional) foods are popular in Taiwan.

The PSQI scores and the proportion of individuals with poor sleep quality were significantly higher in the northern area than in other areas. A cross-sectional survey was conducted among community-dwelling elderly in northern Taiwan between March 2009 and November 2009 and found that as much as 41 % of individuals had sleep disorder [34] although the study population was limited to the elderly. A study cited above also found that the prevalence of sleep disorder was higher among individuals who were living in cities with the highest urbanization level [21]. The average age of poor-sleep-quality individuals was still older in the eastern area due to the more aged population, and they were still predominantly female. Of interest, the proportion of hypnotics use among poor-sleep-quality individuals was significantly lower in the northern area, although those individuals had the shortest sleep duration. In contrast, the proportion of poor sleep quality was lowest in the eastern area, but the individuals with poor sleep quality in this area had the longest sleep duration. That may be related to the highest proportion of hypnotics use and the differences in urbanization and culture. However, there were relatively few participants from the eastern area in this study. We still need further studies with larger, randomized samples to confirm this result.

In the analysis of PSQI subscale scores there were significant differences among the four areas in Taiwan, except ‘subjective sleep quality’ and ‘habitual sleep efficiency’. The poor-sleep-quality individuals in the eastern area had more problems with ‘sleep latency’ and ‘use of sleeping medication’; those in the northern area had more problems with ‘sleep duration’ and ‘daytime dysfunction’. The ‘daytime dysfunction’ score was highest in the northern area, which may be related to this area having the shortest sleep duration. It may be that different local conditions and customs in different areas lead to different sleep problems.

It is important to know the status of sleep quality because there are many co-morbid conditions that can present with sleep problems, such as depression and anxiety, as well as chronic medical conditions. Co-morbid conditions have a bidirectional relationship with sleep disorder, with each influencing or exacerbating the other and requiring concurrent assessment and management. Although the study design was not a randomized sampling method to examine the prevalence and incidence of sleep disorder in Taiwan, it provided the population-based and updated information of sleep quality coming from four parts, northern, central, southern, and eastern part of Taiwan, to have its overall examination for sleep quality and reported the frequency of hypotonic drugs used. Besides, the same PSQI [27, 35], a validated sleep screening questionnaire in primary care, was used to screen the sleep quality in Taiwan. In future, we may consider adding this validated sleep screening questionnaire to the routine health examination (i.e.: civil servant, workers, the elderly…etc.) to understand the sleep situation of different populations and even apply it to other countries to have the homogeneous results to compare.

There are several limitations in this study. First, the participants were not recruited using randomized sampling from the general population, such that the results may not be representative of the entire Taiwanese population. Second, we had no detailed information about co-morbidities and education and no information on any further diagnosis to clarify the subtype of sleep disorder. However, the PSQI has been validated with reliable sensitivity and specificity in screening sleep quality, and this study was focused on screening, not diagnosing. Third, the prevalence of poor sleep quality may be under-estimated because only physically independent people could join the “walk-in” screening program. Finally, the design of this study was cross-sectional, which consequently ruled out a reliable separation of primary sleep complaint from sleep complaint secondary to another disorder or condition. Thus, a large-scale longitudinal study is needed to identify the relationship between sleep problems and its many co-morbidities and correlates, ex.: depressed disorder.

## Conclusion

This study found a high ratio of poor sleep quality among Taiwanese, especially in the northern area, and about one-fifth of poor-sleep-quality individuals had used hypnotics in the past 4 weeks to help fall asleep, with the highest ratio in the eastern area. Sleeping quality is a crucial issue in Taiwan.

## Abbreviations

MPC: Mentality Protection Center
PSQI: Pittsburgh Sleep Quality Index
AD8: Ascertainment of dementia 8
CES-D: Center for Epidemiologic Studies-Depression
PSQI-T: the Taiwanese version of the Pittsburgh Sleep Quality Index
IRB: Institutional Review Board

## References

[1] Sateia MJ (2014). International classification of sleep disorders-third edition: highlights and modifications. Chest.

[2] Ohayon MM, Reynolds CF (2009). Epidemiological and clinical relevance of insomnia diagnosis algorithms according to the DSM-IV and the International Classification of Sleep Disorders (ICSD). Sleep Med.

[3] Uhlig B, Engstrøm M, Ødegård S, Hagen K, Sand T (2014). Headache and insomnia in population-based epidemiological studies. Cephalalgia.

[4] Roth T. Insomnia: definition, prevalence, etiology, and consequences. J Clin Sleep Med. 2007;3:S7–10. PMC1978319 17824495

[5] Britt H MG, Charles J, Henderson J, Bayram C, Pan Y, Valenti L, Harrison C, O’Halloran J, Fahridin S. General practice activity in Australia 2009–10. Canberra: Australian Institute of Health and Welfare, 2010; 2010.

[6] Baglioni C, Battagliese G, Feige B, Spiegelhalder K, Nissen C, Voderholzer U, Lombardo C, Riemann D (2011). Insomnia as a predictor of depression: a meta-analytic evaluation of longitudinal epidemiological studies. J Affect Disord.

[7] Riedel BW, Lichstein KL (2000). Insomnia and daytime functioning. Sleep Med Rev.

[8] Strine TW, Chapman DP (2005). Associations of frequent sleep insufficiency with health-related quality of life and health behaviors. Sleep Med.

[9] Chen X, Beydoun MA, Wang Y (2008). Is sleep duration associated with childhood obesity? A systematic review and meta-analysis. Obesity.

[10] Vgontzas AN, Liao D, Pejovic S, Calhoun S, Karataraki M, Bixler EO (2009). Insomnia with objective short sleep duration is associated with type 2 diabetes A population-based study. Diabetes Care.

[11] Vozoris NT (2013). The relationship between insomnia symptoms and hypertension using United States population-level data. J Hypertens.

[12] Fullerton D (2006). The economic impact of insomnia in managed care: a clearer picture emerges. Am J Manag Care.

[13] Ministry of Health and Welfare,Statistics,. http://www.mohw.gov.tw/EN/Ministry/Statistic.aspx?f_list_no=474. Accessed 2015 July 28.

[14] Nomura K, Yamaoka K, Nakao M, Yano E (2005). Impact of insomnia on individual health dissatisfaction in Japan, South Korea, and Taiwan. Sleep.

[15] Kao CC, Huang CJ, Wang MY, Tsai PS (2008). Insomnia: prevalence and its impact on excessive daytime sleepiness and psychological well-being in the adult Taiwanese population. Qual Life Res.

[16] Susan Shur-Fen GAU (2006). Prevalence of sleep problems and their association with inattention/hyperactivity among children aged 6–15 in Taiwan. J Sleep Res.

[17] Huang YS, Wang CH, Guilleminault C (2010). An epidemiologic study of sleep problems among adolescents in North Taiwan. Sleep Med.

[18] Chen HC, Su TP, Chou P (2013). A nine-year follow-up study of sleep patterns and mortality in community-dwelling older adults in Taiwan. Sleep.

[19] Chueh K-H, Yang M-S, Chen C-S, Chiou S-M (2009). Poor sleep quality and alcohol use problems among elderly Taiwanese aboriginal women. Int Psychogeriatr.

[20] Chen W-C, Lim P-S, Wu W-C, Chiu H-C, Chen C-H, Kuo H-Y, Tsai T-W, Chien P-I, Su Y-J, Su Y-L (2006). Sleep behavior disorders in a large cohort of Chinese (Taiwanese) patients maintained by long-term hemodialysis. Am J Kidney Dis.

[21] Hsu YW, Ho CH, Wang JJ, Hsieh KY, Weng SF, Wu MP (2013). Longitudinal trends of the healthcare-seeking prevalence and incidence of insomnia in Taiwan: an 8-year nationally representative study. Sleep Med.

[22] Yang YH, Galvin JE, Morris JC, Lai CL, Chou MC, Liu CK (2011). Application of AD8 questionnaire to screen very mild dementia in Taiwanese. Am J Alzheimers Dis Other Demen.

[23] Radloff LS (1991). The use of the center for epidemiologic studies depression scale in adolescents and young adults. J Youth Adolesc.

[24] Buysse DJ, Reynolds CF, Monk TH, Berman SR, Kupfer DJ (1989). The Pittsburgh Sleep Quality Index: a new instrument for psychiatric practice and research. Psychiatry Res.

[25] Buysse DJ, Reynolds Iii CF, Monk TH, Berman SR, Kupfer DJ (1989). The Pittsburgh sleep quality index: a new instrument for psychiatric practice and research. Psychiatry Res.

[26] Buysse DJ, Reynolds CF, Monk TH, Hoch CC, Yeager AL, Kupfer DJ (1991). Quantification of subjective sleep quality in healthy elderly men and women using the Pittsburgh Sleep Quality Index (PSQI). Sleep.

[27] Tzeng JI, Fu YW, Lin CC (2012). Validity and reliability of the Taiwanese version of the Pittsburgh Sleep Quality Index in cancer patients. Int J Nurs Stud.

[28] Department of Civil affair, Ministry of the Interior, Taiwan. http://sowf.moi.gov.tw/stat/month/elist.htm.

[29] Department of Statistics, Minister of the Interior. Statistical Yearbook of Interior. http://www.moi.gov.tw/stat/english/index.asp. Accessed March 31, 2014.

[30] Busto UE, Ruiz I, Busto M, Gacitua A (1996). Benzodiazepine use in Chile: impact of availability on use, abuse, and dependence. J Clin Psychopharmacol.

[31] De Las Cuevas C, Sanz E, De La Fuente JA, Cabrera C, Mateos A (1999). Prescribed daily doses and ‘risk factors’ associated with the use of benzodiazepines in primary care. Pharmacoepidemiol Drug Saf.

[32] Zandstra SM, Furer JW, van de Lisdonk EH, van’t HM, Bor JH, van Weel C, Zitman FG (2002). Different study criteria affect the prevalence of benzodiazepine use. Soc Psychiatry Psychiatr Epidemiol.

[33] Morin CM, Bélanger L, LeBlanc M (2009). The natural history of insomnia: a population-based 3-year longitudinal study. Arch Intern Med.

[34] Tsou M-T (2013). Prevalence and risk factors for insomnia in community-dwelling elderly in northern Taiwan. J Clin Gerontol Geriatr.

[35] Buysse DJ, Reynolds CF, III, Monk TH, Berman SR, Kupfer DJ. The Pittsburgh sleep quality index: a new instrument for psychiatric practice and research. Psychiatry Research, 28:193–213. 10.1016/0165-1781(89)90047-4 2748771

